# Burnout among physicians

**DOI:** 10.3402/ljm.v9.23556

**Published:** 2014-02-17

**Authors:** Maya Romani, Khalil Ashkar

**Affiliations:** Department of Family Medicine, American University of Beirut, Beirut, Lebanon

**Keywords:** burnout, health care professionals, stress management, mindfulness-based stress reduction programs, physicians well-being

## Abstract

Burnout is a common syndrome seen in healthcare workers, particularly physicians who are exposed to a high level of stress at work; it includes emotional exhaustion, depersonalization, and low personal accomplishment. Burnout among physicians has garnered significant attention because of the negative impact it renders on patient care and medical personnel. Physicians who had high burnout levels reportedly committed more medical errors. Stress management programs that range from relaxation to cognitive-behavioral and patient-centered therapy have been found to be of utmost significance when it comes to preventing and treating burnout. However, evidence is insufficient to support that stress management programs can help reducing job-related stress beyond the intervention period, and similarly mindfulness-based stress reduction interventions efficiently reduce psychological distress and negative vibes, and encourage empathy while significantly enhancing physicians’ quality of life. On the other hand, a few small studies have suggested that Balint sessions can have a promising positive effect in preventing burnout; moreover exercises can reduce anxiety levels and exhaustion symptoms while improving the mental and physical well-being of healthcare workers. Occupational interventions in the work settings can also improve the emotional and work-induced exhaustion. Combining both individual and organizational interventions can have a good impact in reducing burnout scores among physicians; therefore, multidisciplinary actions that include changes in the work environmental factors along with stress management programs that teach people how to cope better with stressful events showed promising solutions to manage burnout. However, until now there have been no rigorous studies to prove this. More interventional research targeting medical students, residents, and practicing physicians are needed in order to improve psychological well-being, professional careers, as well as the quality of care provided to patients.

Burnout is a syndrome seen in demanding jobs and in people who care for others such as social workers, teachers, and healthcare professionals (1).

Healthcare workers, particularly physicians, are exposed to high levels of distress at work. Persistent tension can lead to exhaustion, psychological, and/or physical distress. Moreover, burnout syndrome may increase the risk of medical errors and decrease job satisfaction, which incites early retirement (2–4).

According to Maslach et al., burnout has three interrelated dimensions: emotional exhaustion, depersonalization, and low personal accomplishment. Prolonged exposure to stress is usually the main cause of emotional exhaustion and it manifests through the loss of enthusiasm for work, feeling helpless, trapped, and defeated. Depersonalization occurs when physicians treat patients indifferently, objectify them, and develop a negative attitude toward their colleagues and profession. Inefficiency, or the lack of a sense of personal achievement, is characterized by the individual's withdrawal from responsibilities and detachment from the job (5, 6).

Maslach Burnout Inventory (MBI), the most frequently used questionnaire, includes 22 items that measure all three burnout dimensions. MBI is considered the golden standard for identifying burnout in medical research literature (6).

Multiple studies have indicated a high prevalence of burnout among practicing physicians and have shown that one-third of physicians have experienced burnout at certain points throughout their careers (7). Burnout begins to cultivate its seeds during the medical school days, continues throughout the residency period, and finally matures in the daily life of practicing physicians. Studies suggest that the prevalence of burnout among medical students ranges between 31 and 49.6% (8). Among residents, 50 and 76% of surgical and internal medicine residents are affected, respectively (9). Cohen et al. found that at least one-third of Canadian medical residents from different specialties experience a stressful life (10). On the contrary, Legassie et al. report that 12.5% of medical residents scored positively on the three dimensions altogether (11). Even higher rates were recorded in a study done among Lebanese residents (12).

The burnout rate seems to be even more pronounced among practicing physicians. In a recent study from the United States, 45.8% of physicians reported having at least one symptom of burnout (7). Similarly, the study by the European General Practice Research Network Burnout Study Group, which included 1,400 family physicians in 12 European countries, revealed the following: 43% of respondents scored high for emotional exhaustion, 35% for depersonalization, and 32% for low personal accomplishment, while 12% of participants suffered from burnout in all three dimensions (13). Another study that included more than 500 physicians in the United Kingdom demonstrated that at least one third of the physicians revealed features of burnout (14). These results are comparable to those of similar studies conducted in some Arab countries like Yemen, Qatar, and Saudi Arabia (15–17).

The different outcomes of the aforementioned studies were not affected by the specialty of the practicing physicians, despite the fact that rates were noticeably high among family physicians (13, 18, 16) and general surgeons (3, 14).

Shanafelt concluded that burnout is more likely to occur with trauma surgeons, urologists, otolaryngologists, vascular and general surgeons, and younger healthcare professionals having children in addition to working more than 60 hours per week, having more calls per week than the regular (>2 nights/week), and having compensation determined entirely based on billing (19).

## Intervention methods

Numerous studies suggest that the difficulty that physicians face with balancing their personal and professional lives is a major contributor to distress. To reduce stress at work, one should consider interventions on two levels: the individual and the environment.

### Stress management courses

Stress management ranges from relaxation to cognitive-behavioral and patient-centered therapy. The latter intervention targets organizational and job-related designs (20). Evidence has shown that healthcare providers who seek help or resort to coping and productive strategies tend to experience lower levels of emotional exhaustion than those who do not (21).

Stress reduction programs, focusing on cognitive behavioral techniques, were found to be of utmost significance when it comes to preventing and treating burnout in healthcare professionals.

They can be divided into programs focusing on primary, secondary, and tertiary prevention, where secondary and tertiary interventions focus on specific needs for each target group. Long-term effectiveness of these programs in preventing burnout depends on providing a combination of psychoeducational treatment combined with follow-up booster sessions and on the duration of the program, the focus on the problem, and the sustainability of the supply (22).

Results of systematic reviews that evaluated stress management strategies among general medicine practitioners (GPs) reported that relaxation and cognitive-behavioral skills proved helpful. Moreover, group methods are both more cost-effective and more beneficial than individual counseling (23).

Gardiner and colleagues evaluated the effect of 15 h of stress management training programs on 85 Australian GPs. The programs focused on areas of stress reaction, psychoeducation, relaxation techniques, and cognitive interventions. The work-related stress levels of participants significantly decreased, while their general well-being and quality of life improved over a period of 12 weeks following the course's administration (24).

Two non-randomized controlled trial (non-RCT) intervention studies that included a lecture, research information, and a group discussion with medical students yielded no significant effects on depression, alcoholism, or stress level (25, 26). However, another non-RCT of a 10-session mindfulness-based meditation course improved the overall mood of medical students in the intervention group (27). Skodova showed that sociopsychological training could lessen the level of burnout and positively influence the personality factors that are susceptible to burnout among healthcare students (28).

With residents, Feld et al. found that an intervention program in professional development improved residents’ self-awareness and willingness to explore their feelings. This program consisted of 11 sessions of open discussions and problem solving within a flexible, group-determined set of agenda items (29).

On the contrary, McCue et al. concluded that a single, all-day stress management workshop given to medicine and pediatric residents alleviated their emotional exhaustion for as long as 6 weeks after the intervention (30). Furthermore, a study conducted among family medicine residents showed that their emotional exhaustion had eased as a result of mediation and breathing exercises (31).

*Mindfulness* is defined as a self-directed practice for relaxing the body and calming the mind through focusing on present-moment awareness. The emphasis of mindfulness is staying in the present moment, with a non-judging, non-striving attitude of acceptance. Mindful meditation represents a complementary therapy that has shown promise in the reduction of negative stress and the extraneous factors that lead to burnout. Many studies evaluated these ‘mindfulness-based’ intervention techniques and showed that they potentially play a role in decreasing stress and burnout.

Krasner and colleagues evaluated the effects of an intensive educational program that included mindful meditation, self-awareness exercises, narratives about clinical experiences, appreciative interviews, didactic material, and discussions on primary care physicians. Participants demonstrated improvements in mindfulness, which was correlated with an improvement in their overall mood, empathy (emotional exhaustion), personal accomplishment, and personality over the course period with sustained effects of up to 15 months (32).

Goodman et al. evaluated the training in four types of formal mindfulness practices: body scans, mindful movement, walking, and sitting meditation, as well as discussions focusing on the application of mindfulness in the workplace. MBI scores significantly improved after the course for both physicians and other healthcare providers in the areas of emotional exhaustion, depersonalization, and personal accomplishment. Mental well-being was also enhanced, but there were no significant changes in the physical health scores (33).

Moreover, a group of nurses who were unable to personally join the traditional mindfulness program attended a telephonic session. There was a significant improvement in their general health, while their stress and burnout levels decreased. These developments were sustained for 4 months after the study period (34).

Similarly, Shapiro et al. and Martín-Asuero and colleagues found that mindfulness-based stress reduction interventions efficiently reduce psychological distress and ‘negative vibes’ and encourage empathy while significantly enhancing physicians’ quality of life (35, 36).

Shanafelt concluded that training physicians in the art of mindful practice has the potential to promote physician's health through work (9). Isaksson and colleagues observed that even short-term counseling sessions, either on an individual basis for 1 day or a group basis lasting 1 week, significantly reduces emotional exhaustion among Norwegian doctors (37).

Mindful meditative practice can be a cost-efficient method of improving physicians’ well-being and enhancing their approach to patient-centered care.

In addition to that, a qualitative study showed that music therapy helps physicians relax, rejuvenate, and re-focus, thus enabling them to energetically pull through their shifts. However, no difference in self-reported burnout, sense of coherence, and job satisfaction was noticed (38).

However, two Cochrane reviews concluded that evidence is insufficient to support that stress management programs can help in reducing job-related stress beyond the intervention period in healthcare professionals and little evidence exists in long-term interventions with booster or refresher courses (39).

### Balint sessions

Balint sessions are group sessions that train doctors on how to apply a patient-centered approach with a special focus on doctor–patient relationships. They are known as a common therapeutic strategy that reduces stress and burnout symptoms. Surprisingly, however, interventional studies evaluating the function of Balint groups are scarce (Table 1). Two small studies suggested that Balint sessions may help to prevent stress and burnout, especially among residents (40). A study on general practitioners over 3–15 years found them to have increased job satisfaction after attending Balint therapeutic sessions (41).

**Table 1:** Summary of interventional studies pertinent to burnout

Study	Intervention content	Participants	Duration and post-test F/U	Outcomes
Ball, 2002 (25)	Self-care lecture and group discussion each semester	54 Medical students	1 year	Change in health habits* Emotional and academic performance*
Bourbonnais, 2006 (51)	Change work setting: reduce adverse job psychosocial factors	674 healthcare professionals	2 months F/U: 1 year	Burnout (EE, DP, PA)*
Dunn, 2007 (48)	Data-guided interventions and a systematic improvement process	22 physicians	4 years	Burnout (PA*, EE*, DP§) Quality work competence*
Gardiner, 2004 (24)	Cognitive-behavioral	85 GPs	15 h F/U: 12 weeks	General psychological stress* Quality of work life§ Work-related stress and morale§
Goodman, 2012 (33)	MBSR	93 healthcare professionals	2.5 h/week for 8 weeks; 11 times over 6 years	Burnout (EE*, DP*, PA*) Mental well-being* Physical well-being§
Isaksson, 2008 (37)	Counseling about motivating reflection and acknowledgement of the doctors’ situation and personal needs	185 physicians	1 year	EE*
Kjeldmand, 2008 (41)	Balint sessions	9 GPs	3–15 years	Job satisfaction* Burnout§
Krasner, 2009 (32)	Educational program in mindful communication	70 GPs	8 weeks F/U: 10 months	Mindfulness* Burnout (EE, DP, PA*) Empathy* Mood disturbance*
Margalit, 2005 (42)	1. Bio-psychological teaching 2. Didactic/interactive sessions 3. Balint sessions	102 GPs	12 weeks F/U: 6 months	Patient centered approach* Burnout (EE, DP, PA)*
Martin-Asuero, 2010 (36)	MBSR	29 healthcare professionals	8 weeks F/U: 3 months	Stress-related psychological distress*
McCue, 1991 (30)	Stress management workshop	43 medicine and pediatrics residents	F/U: 6 weeks	Burnout (EE*, DP*, PA§)
Montero-Marin, 2013 (43)	Stretching exercise	67 workers	10 min after working hours for 3 months	Anxiety levels* Burnout (EE§)
Rosenzweig, 2003 (27)	MBSR	140 medical students	10 weeks	Total mood disturbances*
Shapiro, 2005 (35)	MBSR	10 healthcare professionals	8 weeks	Burnout§ Perceived stress*
Skodova, 2013 (28)	Psychosocial training	111 healthcare students	6 months	Burnout*

* Significant improvement (p<0.05).

§ Non-significant improvement (p>0.05).

F/U, follow-up; MBRS, mindfulness-based stress reduction program.

A study evaluated the effect of (‘didactic’) problem-based reading assignments, lectures and discussions in one arm and (‘interactive’) program consisted of reading assignments, lectures and discussions, in addition to role-playing exercises, Balint groups, and one-to-one counseling by a facilitator in the other arm. It found measurable improvement in patient-centered care and self-esteem among general practitioners in both arms and this was more pronounced in the interactive classes; however, both arms failed to decrease burnout scores (42).

### Exercise

A 10-min stretching exercise in the work place has proven to reduce anxiety levels and exhaustion symptoms while improving the mental and physical well-being of healthcare workers (43).

Aerobic exercise is negatively associated with depression (44). It assists in reducing overwhelming stress (45) and improves the biological markers that may intervene between burnout and cardiovascular disease (46).

In a study on 12 physicians, 12 one-hour aerobic sessions were administered for 2 or 3 days weekly to reach the required level of weekly energy expenditure of 17.5 kcal/kg measured by a calories counter. This was found to significantly reduce the participants’ emotional exhaustion and their degree of depersonalization to a lesser extent. However, no significant change was observed with regard to their sense of personal accomplishment (47). This low-cost intervention method is a promising candidate for future studies about the effect of exercise on the well-being of healthcare workers.

Shanafelt also studied the personal health habits and wellness practices among US surgeons and found that physicians who participated in aerobic and muscle-strengthening exercises according to CDC guidelines had high quality of life scores. Surgeons who placed an emphasis on finding meaning in work, focusing on what is important in life, maintained a positive outlook, and embraced a balanced work/life were less likely to get burned out (3).

### Occupational interventions

Few studies focused on organizational interventions as opposed to individual interventions. One study took place in a primary care center where clinic leaders prioritized physician well-being as much as the quality of care. Physicians identified factors that influenced well-being, followed by plans for improvement with accountability, and measured their well-being regularly using validated instruments. The results showed a significant improvement in the emotional and work-induced exhaustion over the study period (48).

Another study lead by an insurance company targeting hospital employees focused on an organizational control of factors that produced stress, found a reduction in medication errors over the study period. A recent review showed that individual-intervention programs are beneficial in reducing burnout in a short term (6 months or less), while a combination of both personal and organizational interventions have longer lasting positive effects (12 months and more) (49).

Melamed et al. argued that treating burnout is a challenging endeavor (46). The success of many interventions may be limited by the fact that later stages of burnout entail physiological changes that are not easily reversed (50).

Consequently, more long-term studies are needed to highlight intervention plans in order to maintain the positive effects mentioned above and to prevent or treat burnout.

## Conclusions

Burnout among physicians is a common serious entity with devastating personal and professional consequences.

Multidisciplinary actions that include changes in the work environmental factors along with stress management programs that teach people how to cope better with stressful events showed promising solutions to manage burnout. However, up until now, there have been no rigorous studies that prove this.

More interventional research targeting medical students, residents and practicing physicians are needed in order to improve psychological well-being, professional career enjoyment as well as the quality of care provided to patients.
